# Long-term effects of natalizumab on MRI activity and clinical outcomes in Japanese patients with relapsing-remitting multiple sclerosis

**DOI:** 10.1186/s12883-023-03297-1

**Published:** 2023-08-29

**Authors:** Takahiko Saida, Qi Hao, Michihiro Kanda, Yumiko Tani

**Affiliations:** 1 Kansai Multiple Sclerosis Centre, Irino Clinic Inc, TCA Building 4F, 2-3-19 Motomachi, Naniwa-ku, Osaka-shi, Osaka Japan; 2 Biogen Japan Ltd, Nihonbashi 1-chome Mitsui Building 14F 1-4-1, Nihonbashi, Chuo-ku, Tokyo, Japan; 3 Present Address: Kansai Multiple Sclerosis Centre, Kyoto Neurology Clinic, Ukyo-ku, Uzumasa-Yurigamoto-cho 8-32, Kyoto, 616-8144 Japan

**Keywords:** Annual Relapse Rate, Japanese, Magnetic resonance imaging, Natalizumab, Relapsing-remitting multiple sclerosis

## Abstract

**Background:**

Relapsing-remitting multiple sclerosis (RRMS) is the most common phenotype of multiple sclerosis (MS), and its active stage is characterized by active T2 lesions with or without gadolinium (Gd) enhancement on magnetic resonance imaging (MRI). Natalizumab is indicated as monotherapy in adults with active RRMS in Japan. The main objective of this study was to investigate the long-term effect of natalizumab on disease progression in Japanese patients with RRMS using MRI data.

**Methods:**

This retrospective, chart review study was conducted at a single center in Japan. The main study outcome was the yearly proportion of patients with active T2-weighted image lesions detected with or without Gd enhancement on brain MRI (incidence rate) after treatment initiation for up to 5 years. Additional endpoints included annual relapse rate (ARR) and expanded disability status scale (EDSS) score.

**Results:**

This study included data from 85 patients with RRMS who had received natalizumab for ≥ 1 year; of these, 65 (76.5%) were female and the mean ± standard deviation (SD) age at baseline was 37.5 ± 10.0 years. The incidence rate of active T2 lesions was 52.9% (45/85) in the year prior to natalizumab treatment (Year − 1), which decreased to 2.4% and 1.6% in Year 0.5–1.5 and Year 1.5–2.5, respectively. No active T2 lesions were detected in Year 2.5–5.5 in patients who continued natalizumab treatment. EDSS score was stable, improved, and worsened in 61.8%, 26.3%, and 11.8% of patients, respectively. The median (range) EDSS score was 2.0 (0.0–7.0) at baseline (*n =* 85) and remained within a similar range (median score between 1.0 and 2.25 during Years 1–5). ARR decreased from 1.12 relapses per year at baseline to 0.12 relapses per year during Year 1 and remained below 0.15 relapses per year up to Year 5.

**Conclusion:**

The results of this first long-term study evaluating the effect of natalizumab on MRI activity and clinical outcomes in Japanese patients with RRMS suggest that natalizumab markedly reduced disease activity and maintained effectiveness over several years.

**Supplementary Information:**

The online version contains supplementary material available at 10.1186/s12883-023-03297-1.

## Introduction

According to the Multiple Sclerosis International Federation (MSIF), the prevalence of multiple sclerosis (MS) in 2020 varied across countries: European countries had the highest prevalence (133/100,000 people) followed by the USA (112/100,000 people) [1]. In Japan, in 2014, only 19,389 patients with MS or neuromyelitis optica were registered in the National Registry System of the Japanese Ministry of Health, Welfare and Labor [2]. However, the prevalence of MS in Japan has increased in the last several decades, especially in women [3]. The development of MS is known to be influenced by environmental factors. The increased prevalence of MS in Japan may reflect the rapid Westernization of lifestyle, improvement in the sanitary conditions, and decreased frequency of infectious disorders and parasite diseases [4].

The response of Asian patients with MS to disease-modifying therapies (DMTs) may not be the same as that of Western patients; thus, collection of real-world data from Asian MS patients is important to determine if there are any Asian-specific findings. In addition, long-term data from Asian MS patients are very limited for some DMTs due to delays in the launch of new drugs. In particular, natalizumab (Tysabri®, Biogen Idec, Inc., Cambridge, MA) was not launched in most Asian countries until the mid-2010s, and evidence of its use in Asian patients with MS is still scarce.

Magnetic resonance imaging (MRI) plays an important role in the diagnosis and monitoring of MS disease activity and progression by identifying characteristic lesions [1, 5, 6]. Relapsing-remitting MS (RRMS) is the most common phenotype, and in its active stage, presents with new or enlarging T2 lesions with or without gadolinium (Gd) enhancement detected by brain MRI [2]. Japan has the largest number of MRI machines in the world, with 0.047 scanners per 1000 population, followed by the USA (0.035/1000 population) and Italy (0.025/1000 population) [7]. In addition, Japan has a universal health insurance system that provides access to health services and treatment without a gate-keeping process [7], minimizing the cost barriers to MRI that exist in other countries.

Natalizumab, a monoclonal antibody that inhibits leukocyte migration across the blood-brain barrier, reduces central nervous system (CNS) inflammation [8]. Natalizumab monotherapy is indicated in adults with MS in the USA and Europe (approved in 2004 and 2006, respectively) [9, 10], and in Japan (approved 2014) [11]. Although its safety and efficacy, including MRI outcomes, have been demonstrated in Japanese RRMS patients in a phase II clinical trial, the study duration was only 24 weeks. Some longer term evidence is available from an open-label extension of the phase II trial [12, 13] and an interim analysis of a post-marketing surveillance (PMS) study [14]. Despite the effectiveness of natalizumab being best characterized by MRI, neither of these longer-term studies assessed MRI outcomes, which is a common problem of real-world studies due to limited insurance coverage in many other countries.

Due to limited data in Asian patients with RRMS on disease progression according to the number of active MRI brain lesions during long-term natalizumab therapy, more real-world data, including evidence from the start of its approval in Japan in the early 2010s, is needed. To address this gap, we conducted a retrospective chart review study of disease activity and progression with frequent collection of MRI data to detect active lesions in Japanese RRMS patients.

## Methods

### Study design and patients

This retrospective, chart review study was conducted at a single center (Kansai Multiple Sclerosis Centre, Irino Clinic, Osaka) in Japan to investigate the long-term effectiveness of natalizumab in patients with RRMS.

Patients were eligible for inclusion if they were aged > 18 years and had received natalizumab for RRMS for ≥ 1 year by January 2018, with a natalizumab dose interval of up to 12 weeks, and who had disease monitoring with brain MRI (T2-weighted image [WI] MRI and/or Gd-enhanced T1-WI MRI) in the year prior to the first dose of natalizumab and at least once during their treatment. This study included patients who had participated in the phase II Japanese clinical trial of natalizumab (NCT01440101) and its open-label extension (NCT01416155) [12, 13], as well as patients in an ongoing PMS study who had started treatment with natalizumab after its approval [14].

All patients or their proxy (close relative) provided written informed consent prior to participation in the phase II trial, its open-label extension, or the PMS study; only data from consenting patients were included in this chart review.

This study underwent review by the ethics committee of the participating medical institution (Irino Clinic Inc., Osaka City, Osaka, Japan), and was conducted in accordance with the Ethical Guidelines for Medical and Health Research Involving Human Subjects, local regulations, and the Declaration of Helsinki.

Data collected included patient demographics, disease characteristics, brain MRI images (Gd-enhanced lesions and T2 hyperintense lesions), Expanded Disability Status Scale (EDSS) scores, relapse status, and anti-John Cunningham virus (JCV) antibody index. Data for MRI and relapse status were extracted for the year prior to initiating natalizumab (Year − 1) and yearly during natalizumab treatment for up to 5 years. EDSS and anti-JCV antibody index data were collected at baseline and post-natalizumab treatment.

### Outcomes and measures

The main study outcome was to determine the yearly proportion of patients with new and/or enlarging (active) T2-WI lesions detected, with or without Gd-enhanced brain MRI (incidence rate).

Natalizumab had been initiated immediately after stopping previous therapy for RRMS, without drug holidays or wash-out periods. As previous treatment may impact the effect of natalizumab on radiologic measurement, the primary endpoint of the yearly assessment excluded the first 6 months after switching treatment, in order to assess the effect solely of natalizumab. Thus, data were analyzed according to the following schedule: Year 0.5–1.5, included MRI scans between Months 7 and 18 of natalizumab treatment; Year 1.5–2.5 between Months 19 and 30; Year 2.5–3.5 between Months 31 and 42; Year 3.5–4.5 between Months 43 and 54; and Year 4.5–5.5 between Months 55 and 66. Baseline MRI was defined as available MRI scans during Year − 1.

### Additional endpoints

Additional endpoints included the proportion of patients with new brain lesions detected by Gd-enhanced T1-WI MRI; data were grouped by the timing of the MRI scans as previously stated. The number of patients who relapsed, the overall number of relapses per year, and the annual relapse rate (ARR), defined as the total number of relapses experienced each year divided by the number of days treated with natalizumab and multiplied by 365 days, were also investigated. Baseline ARR was calculated based on the number of relapses in Year − 1. Patients’ EDSS scores [15] were determined at baseline, defined as the closest EDSS score measurement prior to natalizumab treatment, and yearly for Years 1–5, defined as EDSS score measured within 4 weeks before or after Week 52 (Year 1), Week 100 (Year 2), Week 148 (Year 3), Week 196 (Year 4), and Week 244 (Year 5).

Anti-JCV antibody index was also monitored throughout the study, at baseline (prior to natalizumab) and during Years 1–5 of treatment. Data were presented according to the number of patients who had an anti-JCV index of ≤ 0.9, 0.9 to ≤ 1.5, or > 1.5, or an anti-JCV status of negative or positive each year, where Year 1 was the measurement at Week 24–48, Year 2 at Week 72–96, Year 3 at Week 120–144, Year 4 at Week 168–192, and Year 5 at Week 216–240. If there was more than one measurement within each time period, the data closest to the reference time point (i.e., Week 48, 96, 144, 192, or 240) was used. Anti-JCV antibodies were detected using the Stratify JCV® two-step enzyme-linked immunosorbent assay (Focus Diagnostics, Cypress, CA, USA) following the protocol described elsewhere [16]. Additionally, this study included post-hoc analyses of the number of active T2-lesions from Month 1 to 6 after initiating treatment with natalizumab and the proportion of patients with no evidence of disease activity (NEDA-3) status in each period (calculated by dividing the number of patients who achieved NEDA-3 [i.e., no increase in EDSS and no clinical and radiological activity] by the total number of patients with sufficient data).

### Statistical analysis

Patient characteristics were summarized using descriptive statistics. Continuous variables were summarized using mean and standard deviation (SD) and categorical variables using count (*n*) and percentages. Changes in ARR over time from baseline were analyzed using negative binomial regression. Data analysis was performed with SAS® Version 9.2, 32-bit edition.

## Results

### Patient disposition and characteristics

This study included data from 85 patients; of these, 65 (76.5%) were female. The mean ± SD age at baseline was 37.5 ± 10.0 years, and 50 patients (58.8%) were aged < 40 years (Table 1). Sixty-six patients (77.6%) had concomitant diseases, with the most common comorbidities (in ≥ 15% of patients) being gastrointestinal disorders (40%; gastric ulcer, gastritis, constipation), nervous system disorders (22%; neuropathy, headache), psychiatric disorders (19%; insomnia), and musculoskeletal and connective tissue disorders (16%; back pain, arthropathy). Before starting natalizumab, 35 patients (41.2%) had been receiving fingolimod, 31 (36.5%) received interferon beta-1a, and four (4.7%) received interferon beta-1b. The mean ± SD disease duration at baseline was 8.7 ± 6.1 years (Table 1).

**Table 1:** Patient demographic and clinical characteristics at baseline

Variable	All patients (*N* = 85)
Sex, *n* (%)	
Male	20 (23.5)
Female	65 (76.5)
Age, years	
Mean ± SD	37.5 ± 10.0
< 40 years, *n* (%)	50 (58.8)
≥ 40 years, *n* (%)	35 (41.2)
Relapses	
Patients with relapses, *n* (%)	56 (65.9)
History of concomitant disease, *n* (%)	66 (77.6)
Previous treatments for MS, *n* (%)	81 (95.3)
Interferon beta-1a	40 (47.1)
Fingolimod	37 (43.5)
Interferon beta-1b	21 (24.7)
Treatment prior to starting natalizumab, *n* (%)	
Fingolimod	35 (41.2)
Interferon beta-1a	31 (36.5)
Interferon beta-1b	4 (4.7)
Disease duration, years (mean [SD])	8.7 (6.1)

MS, multiple sclerosis; SD, standard deviation.

### Natalizumab treatment

This study included 29 patients who had participated in the phase II trial and its open-label extension, and 56 patients who had initiated natalizumab after its launch in Japan.

The mean ± SD and median (range) duration of natalizumab treatment were 2.3 ± 1.1 years and 1.9 (0.9–5.4) years, respectively; 36 patients (42.4%) received natalizumab for ≥ 2 years. Fifty-eight patients discontinued natalizumab; of these, 43 (74.1%) discontinued during the first 2 years of treatment. The main reasons for discontinuation were concerns about progressive multifocal leukoencephalopathy (PML) risk (77.6%) and current or planned pregnancy (12.1%) (Table 2).

**Table 2:** Duration of natalizumab exposure and treatment discontinuation

**Duration of natalizumab exposure** ^a^	***N = 85***
Mean ± SD, duration, years	2.30 ± 1.12
Median (range) duration, years	1.92 (0.9, 5.4)
Duration, *n* (%)	
≤ 1 year	1 (1.2)^b^
> 1 to ≤ 2 years	48 (56.5)
> 2 to ≤ 3 years	17 (20.0)
> 3 to ≤ 4 years	11 (12.9)
> 4 to ≤ 5 years	3 (3.5)
≥ 5 years	5 (5.9)
**Treatment discontinuation**	***n*** **= 58**
Timing of discontinuation, *n* (%)	
≤ 1 year	1 (1.7)^b^
> 1 to ≤ 2 years	42 (72.4)
> 2 to ≤ 3 years	10 (17.2)
> 3 to ≤ 4 years	2 (3.4)
> 4 to ≤ 5 years	2 (3.4)
≥ 5 years	1 (1.7)
Reasons for treatment discontinuation, *n* (%)	
Concerns about the risk of PML	45 (77.6)
Pregnancy and planning	7 (12.1)
Moving and change of hospital	4 (6.9)
Insufficient effect and worsening of disability	1 (1.7)
Use of contraindicated drug	1 (1.7)

^a^Duration from the initiation of natalizumab treatment to data collection or discontinuation. ^b^Exposure (duration from first dose to last dose) of this patient was 344 days (i.e., the patient received natalizumab 12 times).

PML, progressive multifocal leukoencephalopathy; SD, standard deviation.

### Frequency of MRI imaging

MRI scans were performed nearly bimonthly during Year − 1 and Year 0.5–1.5; the mean ± SD number of T2-WI MRI scans was 4.8 ± 3.3 and 6.1 ± 3.7, respectively, and of Gd-enhanced T1-WI MRI scans was 5.1 ± 3.8 and 6.0 ± 3.9, respectively. Subsequently, MRI scans were still performed repeatedly in each patient, with the mean number of both T2-WI and Gd-enhanced T1-WI MRI scans being approximately 4.0, 3.5, 3.0, and 2.0 per year in Years 1.5–2.5, 2.5–3.5, 3.5–4.5, and 4.5–5.5, respectively (Table 3).

**Table 3:** Number of patients with MRI data, and the mean number of MRI scans per year (T2-WI or Gd-enhanced T1-WI)

	Year − 1 (*n* = 85)		Year 0.5–1.5 (*n* = 85)		Year 1.5–2.5 (*n* = 68)		Year 2.5–3.5 (*n* = 26)		Year 3.5– 4.5 (*n* = 11)		Year 4.5–5.5 (n = 8)
T2-WI scans											
Patients, *n* (%)	85 (100)	85 (100)		63 (92.6)		24 (92.3)		9 (81.8)		8 (100)	
Mean ± SD	4.8 ± 3.3	6.1 ± 3.7		4.1 ± 3.0		3.6 ± 2.0		3.1 ± 0.8		2.0 ± 0.5	
Gd-enhanced T1-WI scans											
Patients, *n* (%)	81 (95.3)	83 (97.6)		59 (86.8)		21 (80.8)		8 (72.7)		5 (62.5)	
Mean ± SD	5.1 ± 3.8	6.0 ± 3.9		4.0 ± 3.2		3.5 ± 2.1		2.8 ± 1.0		2.2 ± 0.4	

Year 0.5–1.5 included MRI scans conducted in Months 7–18 of natalizumab; Year 1.5–2.5 in Months 19–30; Year 2.5–3.5 in Months 31–42; Year 3.5–4.5 in Months 43–54; and Year 4.5–5.5 in Months 55–66.

Baseline MRI was defined as available MRI scans from the year prior to initiating natalizumab (Year − 1).

Gd, gadolinium; MRI, magnetic resonance imaging; SD, standard deviation; T1-WI, T1-weighted image; T2-WI, T2-weighted image.

### MRI T2 lesions with or without gd enhancement

**Fig. 1:**
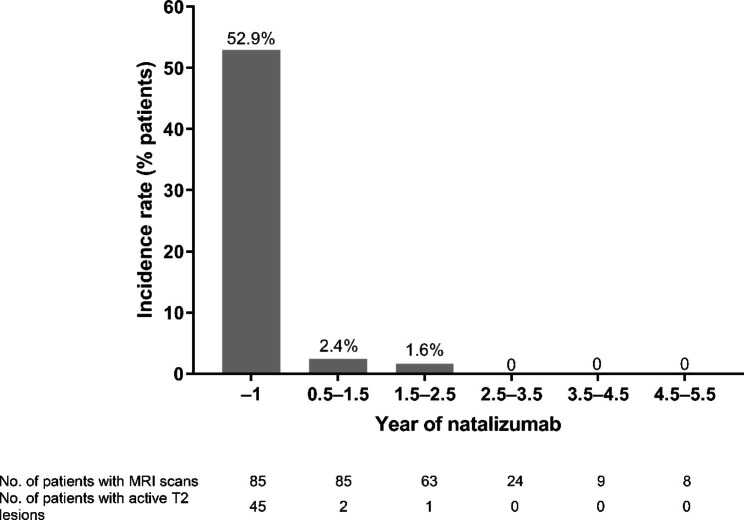
Incidence rate (% of patients) of active (new or enlarged) T2-weighted image lesions. MRI, magnetic resonance imaging

The number of active T2 lesions (i.e., new and/or enlarging T2 lesions) detected with or without Gd-enhanced T1-WI MRI in patients with RRMS treated with natalizumab is summarized in Fig. 1. The incidence rate of active T2 brain lesions was 52.9% (45/85 patients) in Year − 1; of these 45 patients, 41 had lesions with Gd enhancement while four had lesions without Gd enhancement. At Year 0.5–1.5, only two patients (identified here as Patient A and Patient B) had active T2 lesions and, therefore, the incidence rate of active T2 lesions was 2.4% (2/85 patients). During Year 1.5–2.5, of the 63 patients with available MRI data, only one (Patient A) showed active T2 lesions with Gd enhancement (incidence rate of 1.6%). No active T2 lesions were detected in any of the patients with available data subsequently (Fig. 1).

Detailed data on the two poor responders with active T2 lesions are shown in Supplementary Figure S1. In patient A, Gd-enhanced active lesions were continuously present before, during, and after treatment discontinuation. Patient B discontinued treatment because of repeated clinical relapses and worsening disability. The anti-JCV antibody index of Patient A was 2.38 at baseline and 3.23 at 419 days post-dose, while the index of Patient B was 3.22 at baseline and 2.77 at 561 days post-dose. Anti-natalizumab antibody was negative in both cases.

In addition, a post-hoc analysis was performed to assess the MRI data available during the first 6 months of treatment (Supplementary Figure S2). During this time, MRI data were available for all 85 patients and five showed active T2 lesions during Month 1; of these, Gd-enhanced T1-WI lesions were detected in three patients, whereas in two patients, lesions were detected without Gd enhancement. In addition, during Month 2, 14 patients (16.5%) showed active T2 lesions, 13 of whom had active lesions on Gd-enhanced T1-WI MRI. No active T2 lesions or Gd-enhanced T1-WI lesions were detected during Months 3–6.

### EDSS score

**Fig. 2:**
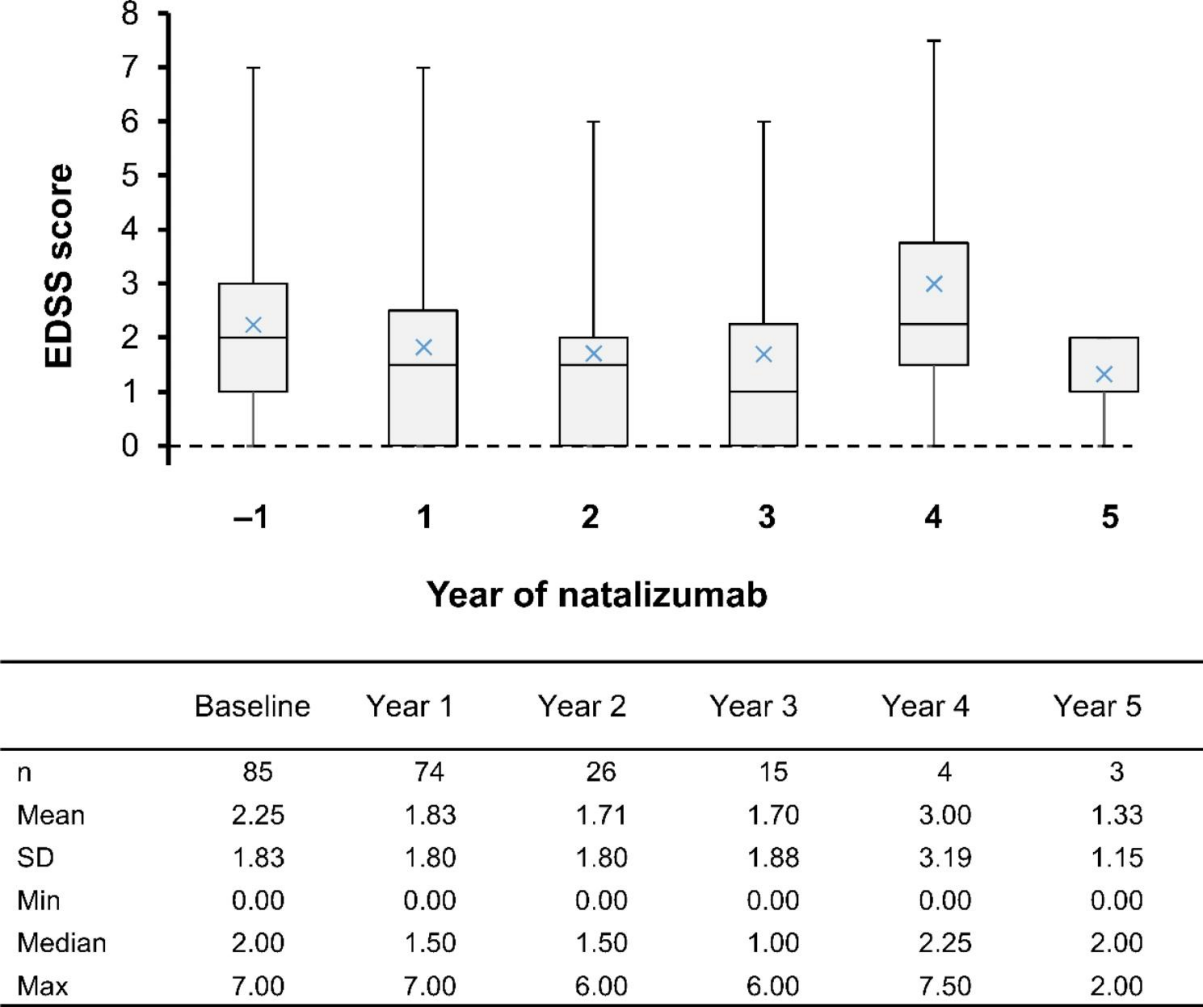
Median EDSS score per year from baseline (Year − 1) to Year 5. Horizontal bar inside each box indicates the median value, the upper and lower whiskers the maximum and minimum values, respectively, and the x symbol indicates the mean value. Years 1, 2, 3, 4, and 5 EDSS scores are those values available within 4 weeks of (before or after) Weeks 52, 100, 148, 196, and 244, respectively. EDSS, Expanded Disability Status Scale; SD, standard deviation

EDSS scores measured at the time point defined for Years 1–5 were available for 74/85 (Year 1), 26/68 (Year 2), 15/26 (Year 3), 4/11 (Year 4), and 3/8 (Year 5) patients; data from patients whose EDSS was collected outside of the assessment time frame were excluded from the analysis. The median (range) EDSS score was 2.0 (0.0–7.0) at baseline (*n* = 85) and remained in a similar range during natalizumab treatment (Fig. 2).

**Fig. 3:**
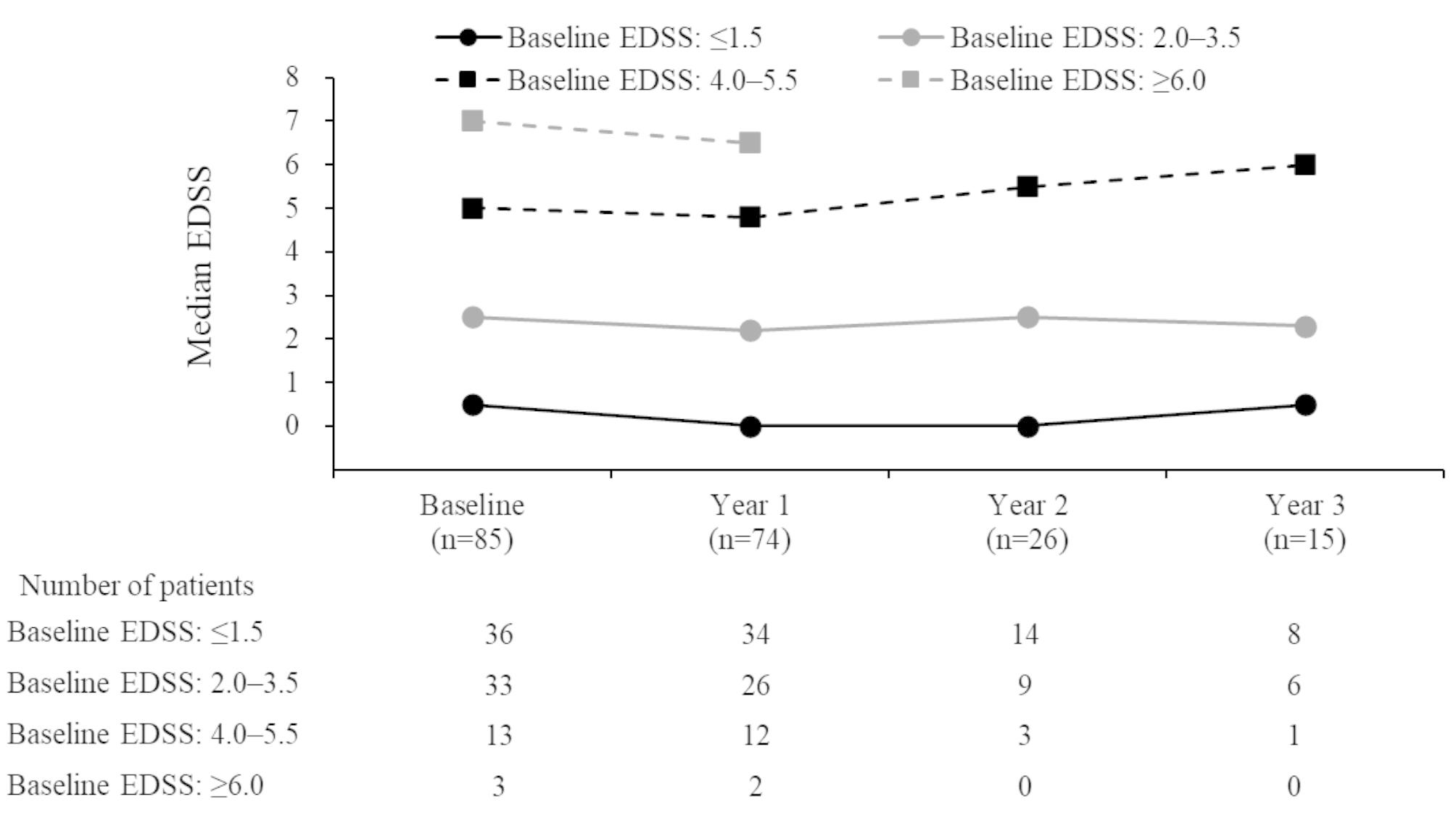
EDSS scores stratified by the baseline EDSS score in patients with relapsing-remitting multiple sclerosis receiving natalizumab. Year − 1 (baseline) EDSS is the closest score available before initiating natalizumab, and Years 1, 2, and 3 are the scores available within 4 weeks (before or after) of Weeks 52, 100, and 148, respectively. EDSS, Expanded Disability Status Scale

Of 76 patients with EDSS scores at baseline and during natalizumab treatment, 61.8%, 26.3%, and 11.8% had stable, improved, and worsened EDSS score at the last assessment time point, respectively. EDSS scores during the first 3 years of treatment, stratified by baseline EDSS score are shown in Fig. 3.

At baseline, the EDSS score was ≤ 1.5 in 36 patients (42.4%) and ≥ 6 in three (3.5%). Patients whose baseline EDSS scores were ≤ 1.5 had median EDSS scores of between 0 and 0.5 during the first 3 years of treatment; those with baseline EDSS scores of 2–3.5 at baseline had median scores of 2.0–2.5 during the first 3 years of natalizumab. The number of patients with baseline EDSS scores of ≥ 4.0 was low after Year 1, mainly due to treatment discontinuation, thus precluding any meaningful analysis of EDSS scores in this group.

### Relapse rate

**Fig. 4:**
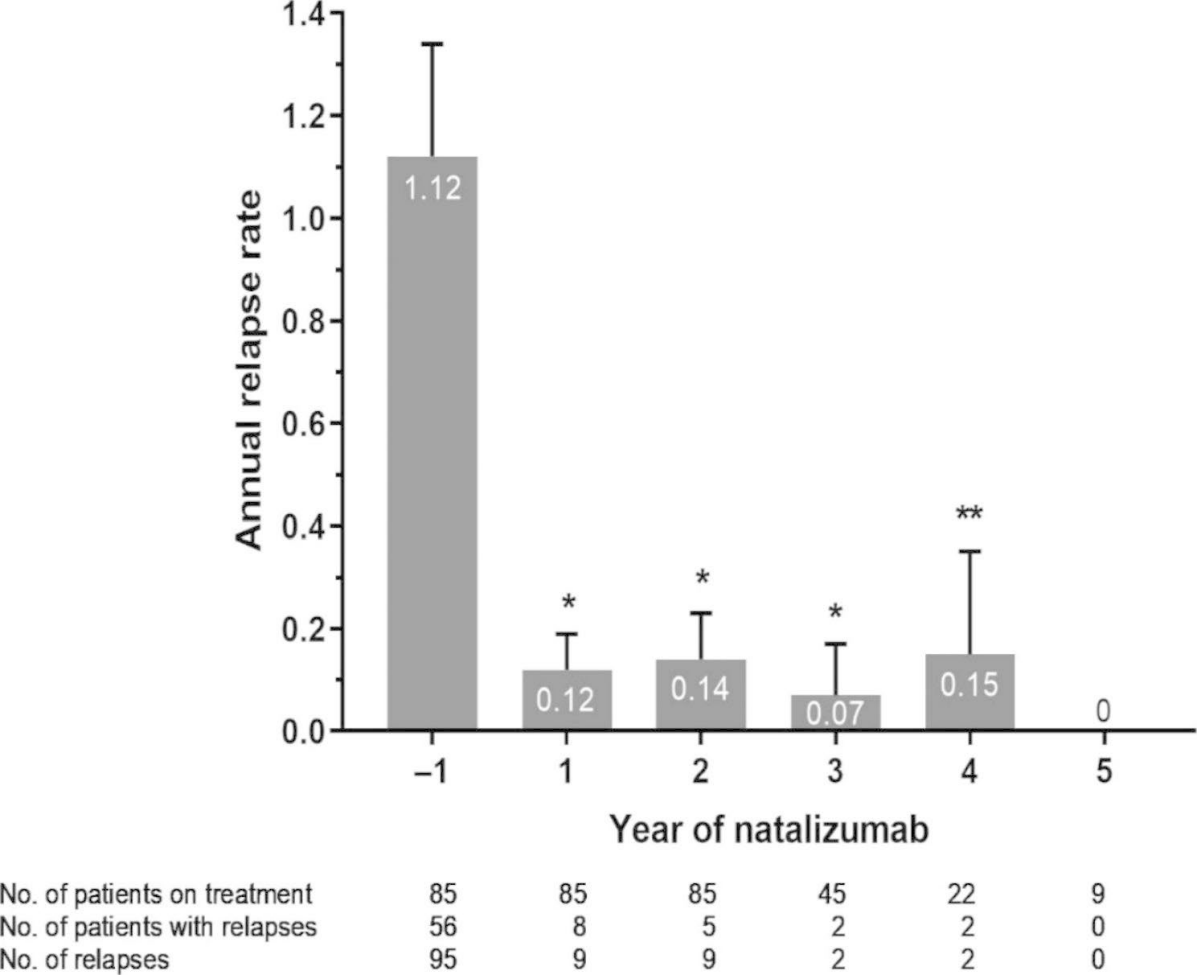
Number of relapses per year from baseline (Year − 1) to Years 1–5. Annual relapse rate was defined as the total number of relapses experienced each year divided by the number of days treated with natalizumab and multiplied by 365 days. *p < 0.0001 vs. baseline; **p = 0.0038 vs. baseline

Relapse data were available for 85 patients at baseline and during Years 1 and 2. The number of patients who relapsed during Year 1 (*n* = 8, 9.4%) and Year 2 (*n* = 5, 5.9%) decreased in comparison with baseline (*n* = 56, 65.9%; Fig. 4). In addition, the total number of relapses also decreased from baseline, from 95 relapses to nine relapses each during Years 1 and 2. The ARR decreased from 1.12 per year at baseline to 0.12 per year during Year 1 and remained below 0.15 per year up to Year 5 (Year 1–3; *p* < 0.0001, Year 4; *p* = 0.0038, Fig. 4).

The proportion of patients who achieved NEDA-3 status was 67.1% (51/76) in Year 1, 71.9% (23/32) in Year 2, and 86.7% (13/15) in Year 3 (NEDA-3 could not be calculated for Years 4 and 5 due to limited EDSS measures).

### Anti-JCV antibody index

Anti-JCV antibody testing became available only after the initiation of the open-label extension of the Japanese phase II trial [12, 13] and, therefore, was used at baseline for only one of the 29 patients included from the clinical trial. Table 4 summarizes the data measured at the timeframe defined in the Methods. Overall, 52 patients had been tested for anti-JCV antibody at baseline, with a negative status rate of 36.5%. Of the 52 patients, 50.0% had an anti-JCV antibody index of ≤ 0.9, 9.6% had an index of > 0.9 and ≤ 1.5, and 40.4% had an index of > 1.5 (Table 4). The percentage of patients with an index of ≤ 0.9 was 63.6% (14/22) at Year 3 and > 70% at later time points.

**Table 4:** Number of patients in each anti-JCV antibody index grouping from baseline (Year − 1) to Year 5 of natalizumab treatment

	Patients in each category in that year, *n* (%)					
	Year − 1 (*n* = 52)	Year 1 (*n* = 53)	Year 2 (*n* = 51)	Year 3 (*n* = 22)	Year 4 (*n* = 12)	Year 5 (*n* = 7)
Anti-JCV antibody index						
≤ 0.9	26 (50.0)	28 (52.8)	23 (45.1)	14 (63.3)	9 (75.0)	5 (71.4)
> 0.9 to ≤ 1.5	5 (9.6)	6 (11.3)	6 (11.8)	1 (4.5)	0	0
> 1.5	21 (40.4)	19 (35.8)	22 (43.1)	7 (31.8)	3 (25.0)	2 (28.6)
Anti-JCV antibody status						
Negative	19 (36.5)	18 (34.0)	14 (27.5)	12 (54.5)	8 (66.7)	4 (57.1)
Positive	33 (63.5)	35 (66.0)	37 (72.5)	10 (45.5)	4 (33.3)	3 (42.9)

Anti-JCV antibody index shown as baseline before natalizumab treatment (Year − 1), Year 1 at 24–48 weeks, Year 2 at 72–96 weeks, Year 3 at 120–144 weeks, Year 4 at 169–192 weeks, and Year 5 at 216–240 weeks. If multiple measurements were taken, the data closest to Weeks 48, 96, 144, 192, and 240 were used.

JCV, John Cunningham virus.

Change in anti-JCV antibody status was assessed in 47 patients who had a JCV antibody test at baseline and at least once during treatment. JCV antibody status remained stable in the majority of the patients; four patients changed status from JCV-negative to JCV-positive, and one patient who was initially JCV-positive converted to negative status (Table 5).

**Table 5:** Change in anti-JCV antibody status from baseline to last examination

Change in JCV antibody status over time	*n* (%)
JCV-negative to JCV-negative	14 (29.8)
JCV-negative to JCV-positive	4 (8.5)
JCV-positive to JCV-negative	1 (2.1)
JCV-positive to JCV-positive	28 (59.6)

JCV, John Cunningham virus

**Fig. 5:**
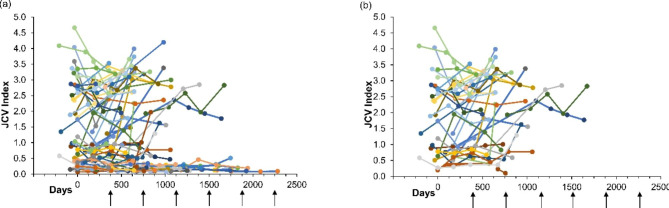
Anti-JCV antibody index per patient in (a) all patients with two or more measurements of JCV antibody index and in (b) patients with two or more measurements of JCV antibody index who discontinued natalizumab due to the risk of PML. Data points at a time point prior to Day 0 are from tests of JCV antibody index prior to baseline. Arrows indicate approximate yearly intervals, from Day 0. JVC, John Cunningham virus; PML, progressive multifocal leukoencephalopathy

The anti-JCV antibody index over time was plotted using data collected from 75 patients who had ≥ 2 tests recorded (Fig. 5a). Overall, 45 patients discontinued treatment due to concerns regarding the risk of developing PML, and 40/45 patients had an anti-JCV antibody index of ≥ 0.9 at any time during monitoring; the anti-JCV antibody index values are plotted in Fig. 5b.

## Discussion

This is the first Japanese study evaluating the long-term effectiveness (up to 5 years) of natalizumab in patients with RRMS using MRI data. Overall, natalizumab markedly reduced the number of active T2-WI and active Gd-enhanced T1-WI lesions.

The incidence rate of active T2 lesions was 52.9% (*n* = 45) in the year prior to natalizumab initiation (baseline) and decreased to 2.4% (*n* = 2) in Year 0.5–1.5 and 1.6% (*n* = 1) in Year 1.5–2.5. Data from the patients who had Gd-enhanced T1-WI lesions showed they were poor treatment responders without neutralizing anti-natalizumab antibodies. These patients continuously showed active lesions, during and after natalizumab treatment, as well as relapses. In contrast, no active T2 lesions were observed in the rest of the patients. The results are in line with a randomized, placebo-controlled clinical trial in a predominantly Caucasian population where up to 2 years of natalizumab treatment significantly reduced the number of active lesions by 83% (*p* < 0.001 vs. placebo) when detected using T2-WI MRI, and by 92% (*p* < 0.001 vs. placebo) when using Gd-enhanced T1-WI MRI. The reduction in the number of lesions in this study was also significant from Year 1 of treatment [17].

The post-hoc analysis of MRI data between Months 1–6 showed that patients had active lesions only in Months 1 and 2, indicating that natalizumab had a fast onset of effect and effectively suppressed lesion accumulation. This result complements and extends data from a previous report in patients undergoing monthly Gd-enhanced T1-WI MRI, which demonstrated marked reductions in the mean number of new lesions in the natalizumab (3 mg/kg) group (0.7/patient) compared with placebo (9.6/patient) (*p* < 0.001) [18].

The EDSS score was stable in 61.8% and improved in 26.3% of patients. Median EDSS score was numerically lower than baseline up to Year 3. At Year 4 and 5, EDSS scores were collected for only 4 and 3 patients, respectively, which possibly explains the apparent increase of median EDSS score. Patients whose EDSS score was low at baseline maintained a similar degree of functionality during long-term natalizumab treatment; in line with a large (*N* = 6148), 10-year, multinational, observational study in RRMS patients treated with natalizumab [19]. Not surprisingly, the study showed that patients with a low EDSS score at baseline (≤ 3) had better functionality over time, with smaller changes in disability progression than patients with higher EDSS scores at baseline (> 3) [19]. Additionally, the STRATA study, which included data from four natalizumab studies in RRMS patients to evaluate its long-term (4.5-year) effectiveness and safety, found that patients who received treatment in an early disease stage had better EDSS scores over time than those who initially received placebo and were switched to natalizumab later [20]. Together with our results, this may indicate that early natalizumab initiation might provide beneficial clinical effects on disability progression in RRMS patients.

In this study, ARR decreased from baseline and remained ≤ 0.15 from Years 1–5 with natalizumab. However, we acknowledge that the sample size, especially from Year 4 onwards, may limit interpretation of our results. Butzkueven and colleagues recently showed that ARR decreased substantially from baseline to Year 1 of natalizumab treatment (ARR = 1.99 to 0.24), and this low ARR was sustained at ≤ 0.20 from Years 2–10 [19].

The proportion of patients with NEDA-3 in this study was 67.1% in Year 1, increasing in Years 2 and 3 to 71.9% and 86.7%, respectively. The relatively low proportion with NEDA-3 in Year 1 was mainly due to the presence of active T2 lesions on MRI in Months 1 and 2 after initiation of natalizumab treatment. This finding appears to be consistent with previous clinical studies of natalizumab, which also showed an increase in the proportion of patients with NEDA-3 over time [21, 22].

Long-term use of natalizumab has been associated with an increased risk of PML, a potentially fatal viral disease caused by the JCV [23]. Ho and colleagues showed that, over 6 years of natalizumab treatment, the cumulative risk of PML was lowest in patients with an anti-JCV antibody index of ≤ 0.9 (0.2%; 1.6 per 1000 patients); however, the cumulative risk increased to 0.9% (8.5 per 1000 patients) in patients with an anti-JCV antibody index of ≥ 0.9 and ≤ 1.5 [23]. Therefore, guidelines advise caution when initiating natalizumab treatment in patients with an anti-JCV antibody index of > 0.9, and emphasize that the risk of developing PML increases when the anti-JCV antibody index reaches values > 1.5 in patients treated with natalizumab for ≥ 2 years [24]. In our study, 45 patients discontinued natalizumab treatment due to concerns about developing PML and of these, 40 patients had an anti-JCV antibody index of > 0.9 at least once during treatment. In 2019, a retrospective analysis of the TOUCH registry showed a lower risk of PML in patients treated with natalizumab extended-interval dosing (EID), compared with the approved dosing interval [25]. Our study includes data collected before the TOUCH analysis and, therefore, does not reflect clinical practice with EID, which may partly explain the high rate of discontinuation due to concerns about PML. Patients with a high baseline anti-JCV antibody index tended to have fluctuating anti-JCV antibody levels, whereas patients with a baseline anti-JCV antibody index of ≤ 0.5 tended to have more stable levels. This fluctuating effect was previously observed in a longitudinal study in patients with MS who received natalizumab for up to 18 months, where a higher anti-JCV antibody index predicted more fluctuation than a negative or low index, which remained negative or consistently ≤ 0.9 [26]. No PML was reported in our study; however, surveillance data from Japan show that four patients have developed PML after natalizumab treatment, one each in 2016 and 2017 and two in 2021 (Biogen, Data on file).

While this study provides the first long-term (5 years) MRI data in RRMS patients in Japan receiving natalizumab, firm conclusions regarding its effectiveness beyond 2–3 years were limited by small patient numbers. Given the rarity of MS in Japan, and the scarcity of data in treated patients for an extended period, our results, nevertheless, provide some insight into active lesion development and patient disability over the long-term. Other study limitations include those specific to its retrospective nature (including attrition bias), and the lack of a comparator. Finally, the EDSS results should be interpreted with caution when assessing the effectiveness of natalizumab; relatively low stable scores may obscure long-term disease worsening (‘silent progression’) in patients with RRMS [27].

## Conclusions

This is the first long-term study evaluating the effectiveness of natalizumab using MRI data in Japanese patients with RRMS, in which early treatment with natalizumab markedly reduced disease activity and maintained effectiveness over several years.

### Electronic supplementary material

Below is the link to the electronic supplementary material.

Supplementary Material 1

## Data Availability

The data generated from this article are available from the corresponding author, upon reasonable request.

## Abbreviations

*EDSS*: Expanded disability status score
*ARR*: Annual relapse rate
*CNS*: Central nervous system
*DMT*: Disease-modifying therapy
*EID*: Extended-interval dosing
*Gd*: Gadolinium
*JCV*: John Cunningham Virus
*MRI*: Magnetic resonance imaging
*MS*: Multiple sclerosis
*MSIF*: Multiple Sclerosis International Federation
*NEDA-3*: No Evidence of Disease Activity – 3 component
*PML*: Progressive multifocal leukoencephalopathy
*PMS*: Post-marketing surveillance
*RRMS*: Relapsing-remitting multiple sclerosis
*SD*: Standard deviation
*WI*: Weighted image
