# Supplementary material for: Long-term effects of natalizumab on MRI activity and clinical outcomes in Japanese patients with relapsing-remitting multiple sclerosis
Source: BMC Neurol. 2023 Aug 29;23:311. doi: 10.1186/s12883-023-03297-1 (PMC10463665; doi:10.1186/s12883-023-03297-1)

**Supplementary Materials**

**Supplementary Figure S1.** Change in magnetic resonance imaging (MRI) activities in two poor responders (Patient A and Patient B) to natalizumab. Both T2-WI and gadolinium (Gd)-enhanced T1-WI lesion activity before baseline, at baseline (Day 0), during natalizumab treatment, and at follow-up assessments are illustrated. Open circle, patient not treated with natalizumab; filled circle, patient treated with natalizumab. T1-WI, T1-weighted image; T2-WI, T2-weighted image.

**
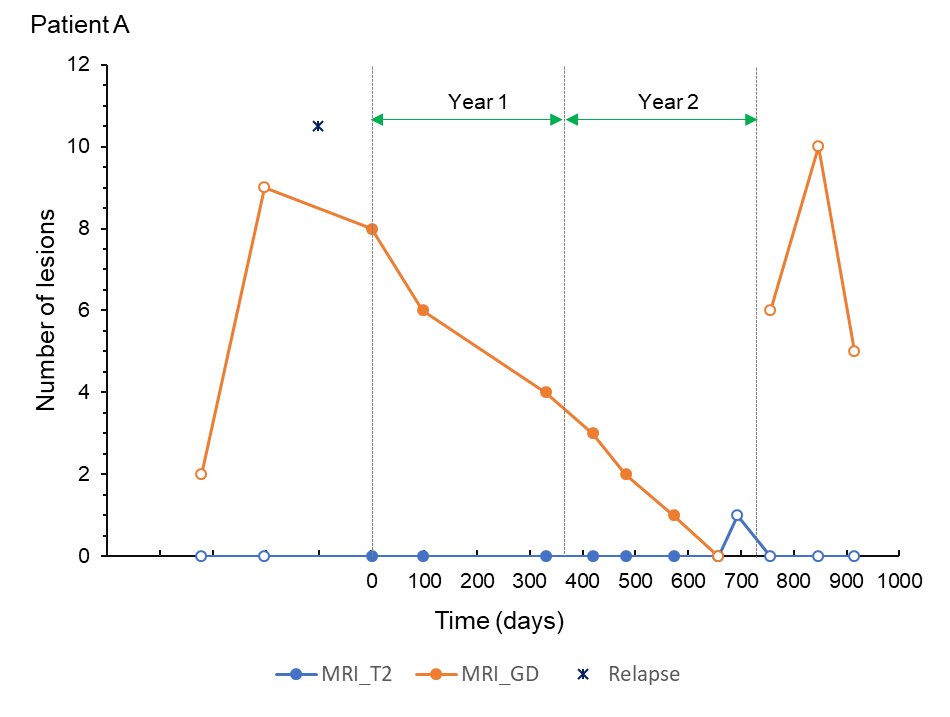
**


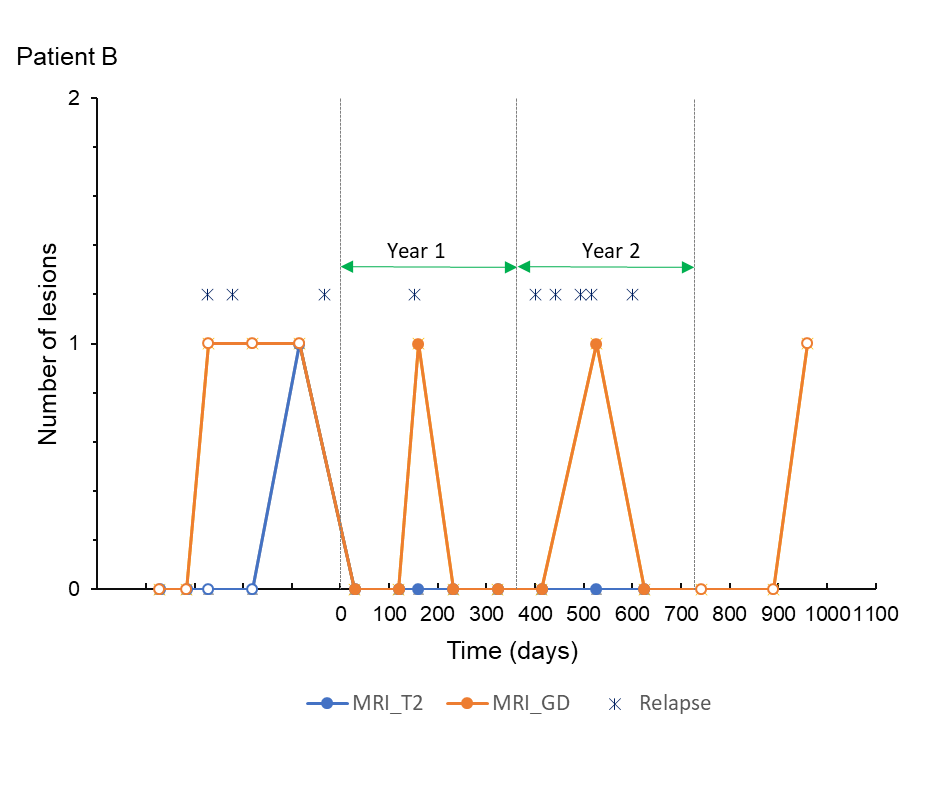


**Supplementary Figure S2.** Magnetic resonance imaging (MRI) activities within 6 months after the initiation of natalizumab treatment. The proportion of patients with active T2-WI lesions with or without gadolinium (Gd)-enhanced MRI. The value above each column is the overall proportion of patients with active lesions (i.e. the sum of those with and without Gd-enhanced lesions). All 85 patients were included in this post-hoc analysis. There were no patients with active lesions at Months 3–6. T2-WI, T2-weighted image.


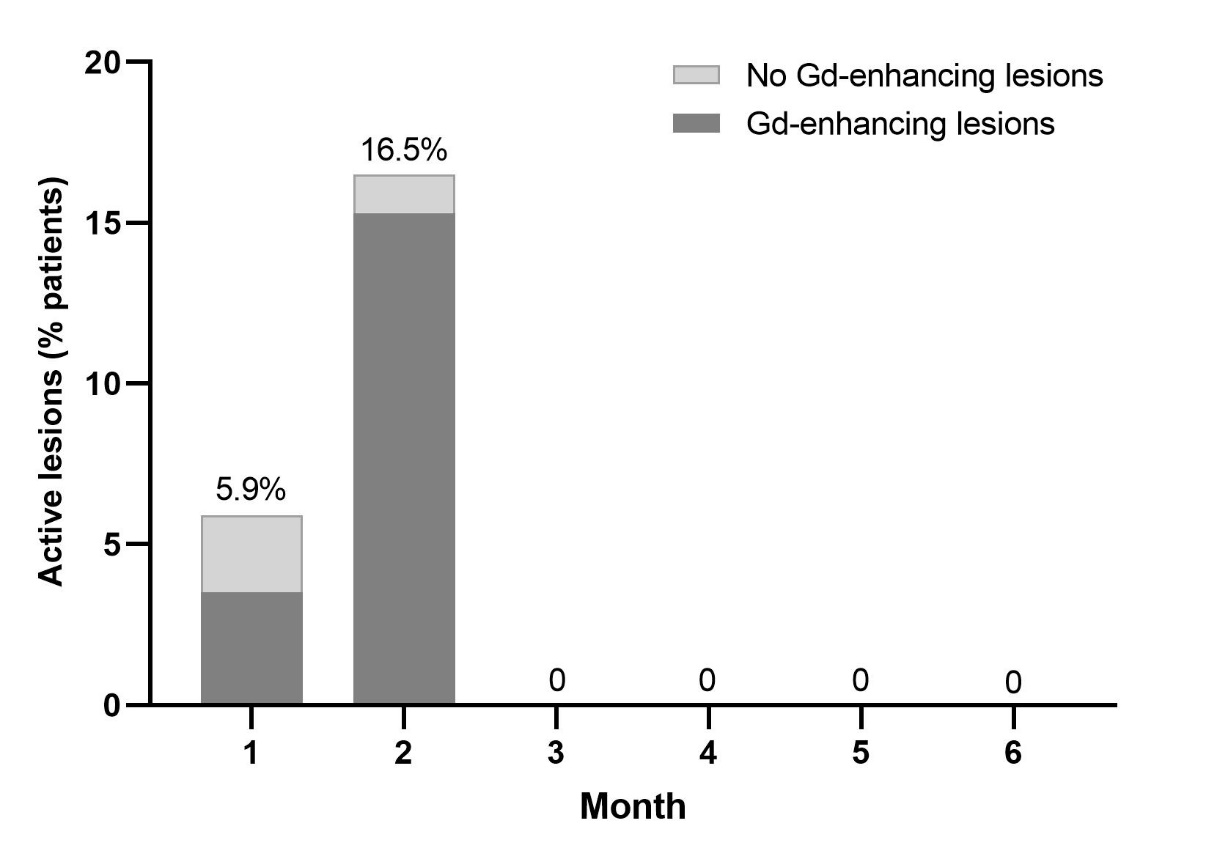

Supplement: Supplementary file 1 — Supplementary Material 1 [file 12883_2023_3297_MOESM1_ESM.docx]
